# Rebuttal letter on behalf of all authors in response to the Letter to the Editor

**DOI:** 10.1016/j.tjpad.2025.100136

**Published:** 2025-04-11

**Authors:** Marwan Noel Sabbagh

**Affiliations:** Department of Neurology, Barrow Neurological Institute, 240W. Thomas Rd, Ste 301, Phoenix, AZ 85013, United States

Dear Dr Gauthier and JPAD Editors,

This is in response to “Letter to the Editor regarding blarcamesine clinical trial published in JPAD vol 12 issue 1.” sent by Jesse Brodkin. We are transparent about all aspects of our work and are providing the responses below.

Please note the provided explicit disclosed conflicts of interest of Jesse Brodkin: “**Previous short stock position in the Sponsor**” (i.e. Anavex Life Sciences). This would question the scientific merits for this Letter to the Editor. Please find below the responses to the three items, which are the content of the Letter to the Editor:

1. The characterization of the findings as robust.
  *This comment demonstrates lack of scientific knowledge appropriate for the interpretation of the published results. The plots Mr Brodkin presented are misleading. The CDR-SB plot of lecanemab cannot be used to compare to the ADAS-Cog13 plot of blarcamesine. The plots were from different companies, different drugs and from different programs, particularly with different tipping point shift increment. We don't know which increment was used in the Letter. Different increments would generate different plots for the same data. The observed difference for blarcamesine ADAS-Cog13 tipping point of −1.973, is close to the significant difference in the MMRM result, −2.027 (p value=0.008). The conclusion provided in the publication in the supplemental Table 7. stands: “This result supports the robustness of the MAR assumption in the primary analysis.”*
2. An inconsistent history of reporting results by the sponsor, Anavex Life Sciences.
  *It is not uncommon that final data of clinical studies could slightly deviate from initial top-line data. Especially since the AD-004 study included updated biomarker data that corrected a few missing data with new technology. However, the provided scores are all consistent and within a narrow margin and reach the same efficacy conclusion.*
3. The failure to make the protocol and statistical analysis plan available to readers.
  *There is no failure to make the protocol and statistical analysis plan available to readers. Manuscript submissions to JPAD have no mandatory requirement for these documents. Since the Sponsor is currently in regulatory submission stage of oral blarcamesine for Alzheimer's disease these documents need to stay proprietary and confidential. Market Authorization Application (MAA) was submitted to EMA in November 2024 and the full submission was accepted in December 2024 by EMA. Submissions to other jurisdictions are following.*

In summary, all questions have been answered and have no scientific merit. Again, this Letter to the Editor follows the same pattern as the previous email inquiries sent by Jesse Brodkin to disparage the Sponsor and JPAD. Specifically, hedge funds target companies to disparage them and their scientific work within respected papers in order to gain financially by shorting them, which is illegal and criminal.

Sincerely,

Marwan Noel Sabbagh

## CRediT authorship contribution statement

**Marwan Noel Sabbagh:** Writing – original draft, Writing – review & editing.

